# Mechanistic Insights into Cs-Ion Exchange in the Zeolite Chabazite from *In Situ* Powder X-Ray Diffraction

**DOI:** 10.1021/acs.jpcc.4c02145

**Published:** 2024-05-29

**Authors:** Daniel S. Parsons, Antony Nearchou, Ben L. Griffiths, Sharon E. Ashbrook, Joseph A. Hriljac

**Affiliations:** † Diamond Light Source Ltd., Harwell Science and Innovation Campus, Didcot OX11 0DE, Oxfordshire, U.K.; ‡ School of Chemistry, University of Birmingham, Edgbaston, Birmingham B15 2TT, West Midlands, U.K.; § School of Chemistry, EaStCHEM and Centre of Magnetic Resonance, North Haugh, University of St Andrews, St Andrews KY16 9ST, Fife, U.K.

## Abstract

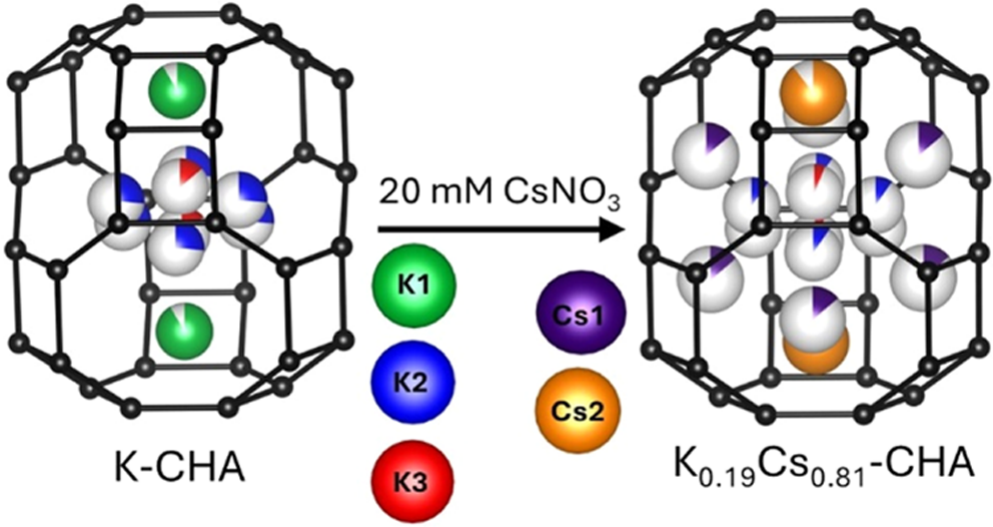

Zeolites contain extraframework cations that are exchangeable under favorable aqueous conditions; this is the fundamental feature for their application in water purification and necessary to produce cation forms for other applications such as catalysis. Optimization of the process is common, but there is little fundamental understanding based on real-time experiments of the mechanism of exchange for most zeolites. The sodium and potassium forms of zeolite chabazite selectively uptake Cs^+^ by ion exchange, leading to its application in removing radioactive ^137^Cs^+^ from industrial nuclear waste streams, as well as from contaminated environments in the aftermath of the Fukushima and Three Mile Island accidents. In this study, *in situ* synchrotron powder X-ray diffraction patterns have been collected on chabazite as it undergoes Cs-ion exchange. Applying Rietveld refinement to these patterns has revealed the time-resolved structural changes that occur in the zeolite as exchange progresses, charting the changes in the spatial distribution of the extraframework cations and water molecules in the structure during the reaction. Ultimately, a detailed mechanistic understanding of how this dynamic ion-exchange reaction occurs has been obtained.

## 1 Introduction

Zeolites are crystalline aluminosilicates composed of corner-shared silicate and aluminate tetrahedra that form regular pores and cages of molecular dimensions. The presence of aluminum renders the framework anionic, so charge-balancing extraframework cations are present within the hydrated channels and cages of the zeolite structure.^1,2^ Aqueous ion-exchange reactions may be observed under favorable conditions between cations in solution and the extraframework cations within the zeolite.^2^ Several industrially important applications exploit ion exchange in zeolites, including water softening, the preparation of catalysts, and the sequestration of radioactive cations, such as ^137^Cs^+^ and ^90^Sr^2+^, from aqueous nuclear waste.^2^ Although optimization of the ion-exchange process via empirical means is ubiquitous, a detailed mechanistic understanding is rare.

^137^Cs (*t*_1/2_ = 30.1 years) is a radionuclide produced in significant quantities as a nuclear fission product of U-235 during nuclear energy generation; the fission yield is 6.22%.^3^ The highly mobile nature of aqueous cesium, coupled with a relatively long half-life, renders the isotope one of the most prevalent and detrimental when accidents occur that contaminate the environment with nuclear waste.^4,5^ In remediation efforts following both the Three Mile Island and Fukushima accidents, the zeolite chabazite was employed to remove cesium from contaminated aqueous environments, as chabazite demonstrates high selectivity in adsorbing cesium by ion exchange in the presence of competing cations such as sodium.^6,7^

**Figure 1:**
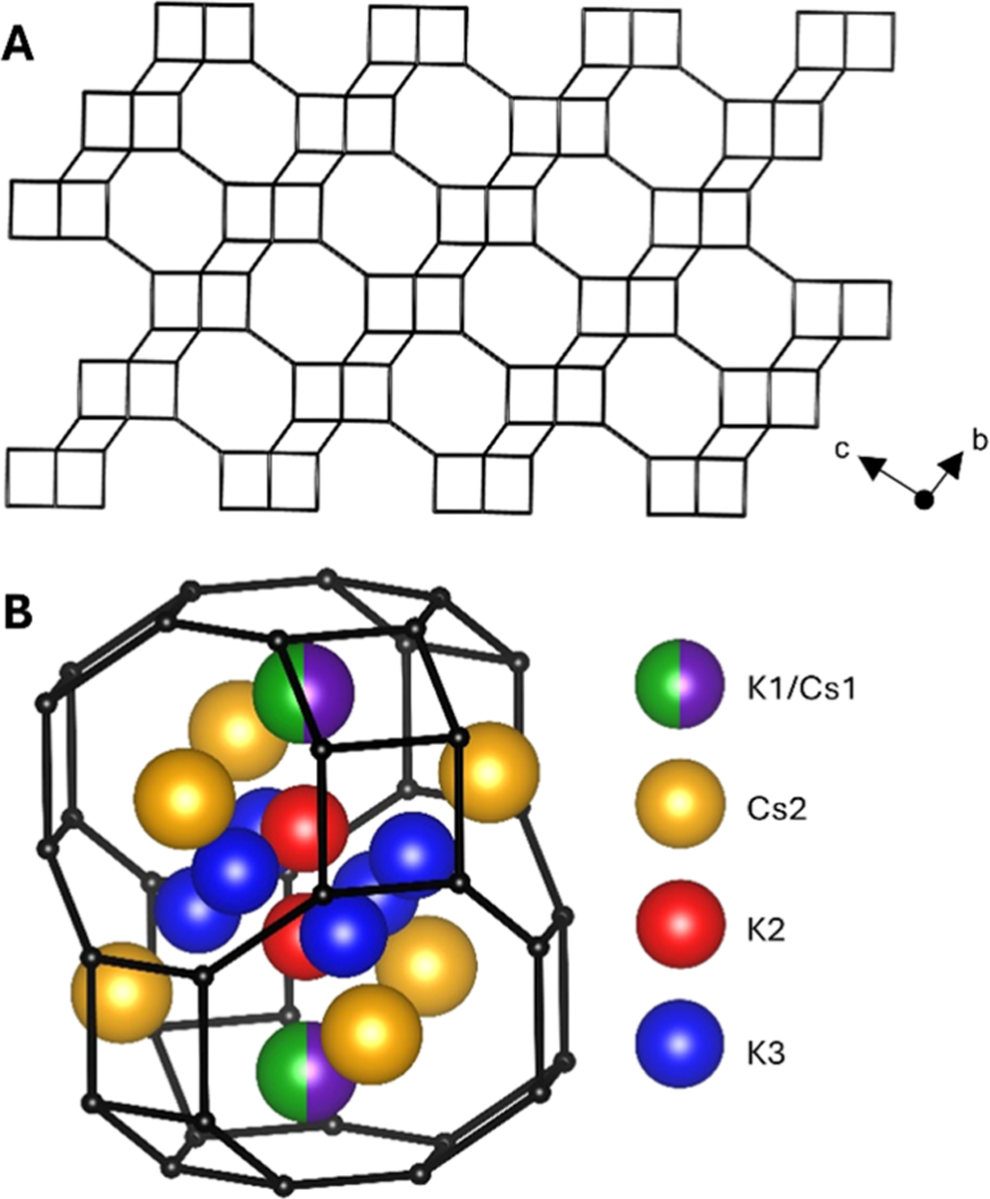
(A) Chabazite lattice fragment showing the stacking of double-6-rings (D6Rs), where each vertical black line joins a Si or Al ion at the center of a [SiO_4_] or [AlO_4_] tetrahedron. (B) Depiction of a chabazite cage showing the cation sites reported by Kong et al.^8^ as labeled.

The zeolite chabazite crystallizes in the rhombohedral space group *R*3̅*m* (No. 166) with a structure comprising double-6-ring (D6R) motifs organized in a repeating ABC stacking sequence as shown in Figure 1. The D6R layers give rise to large periodic cavities termed chabazite cages, in which each contain six single-8-membered ring (S8R) apertures, permitting diffusion of extraframework species between adjoining cages.^8,9^ The crystallographic positions occupied by hydrated extraframework cations, such as K^+^ and Cs^+^, within the chabazite cages at ambient equilibrium conditions are well established when the material contains exclusively one type of cation.^8,9^ There are 3 crystallographic sites that potassium may occupy in the chabazite structure, which are shown within a chabazite cage in Figure 1. The K1 site is positioned within the chabazite cage above the D6R, which forms the base of the cage, and below the D6R, which forms the top of the cage, whereas K2 and K3 reside near the center of the cage. Cesium ions may occupy two crystallographic sites: Cs1, which is analogous to the K1 site, and Cs2, which occupies the center of the S8R apertures, which adjoin neighboring chabazite cages in the structure.^8,10^

Powder X-ray diffraction (PXRD) has only been employed to glean structural insights on inorganic materials during ion exchange in a few reported studies and never before on a zeolite. Cs-ion exchange has been studied by *in situ* methods in crystalline silicotitanate (H–CST)^11,12^ and a zirconosilicate umbite^13^ to reveal changes in the extraframework cation occupancies with time. In these studies, a 1 min data collection was recorded every 140 or 150 s, and the resulting PXRD patterns were analyzed by Rietveld analysis, but only the extraframework cation occupancies were refined, with the atomic positions and thermal displacement parameters for all atoms fixed in all refined data sets, leading to a significant reduction in the quality of fits over the entire data set.^11^

In this present study, Cs-ion exchange has been studied in zeolite chabazite. Owing to the capabilities of beamline I11, at Diamond Light Source, PXRD patterns have been recorded every 20 s, giving significantly improved temporal resolution compared with the previously mentioned *in situ* studies. Moreover, the data quality has permitted detailed Rietveld analysis of the PXRD patterns in which atomic positions and thermal displacement parameters for all atoms in the structure may be refined, as well as the fractional occupancies for both the extraframework cations and water molecules. This has produced a comprehensive time-resolved mechanism for the ion-exchange reaction between aqueous Cs^+^ and the zeolite chabazite.

## 2 Experimental Methods

### 2.1 Synthesis

Potassium chabazite (K-CHA) was synthesized following a modification of the International Zeolite Association verified synthesis.^14^ A 26.8 mL portion of 8.02 M potassium hydroxide stock solution was diluted with deionized water (198 mL), forming a 0.96 M KOH solution (224.8 mL). Ammonium zeolite Y (25 g), obtained from Alfa Aesar, was added to the 0.96 M KOH solution in a polypropylene bottle and shaken manually for 30 s, before being heated in a conventional oven for 96 h at 95 °C. The resulting potassium chabazite was recovered by vacuum filtration, washed copiously with deionized water, and dried at 60 °C.

### 2.2 Thermogravimetric Analysis – Mass Spectrometry (TGA-MS)

The water content of potassium chabazite was determined by thermogravimetric analysis (TGA) with a Netzch STA 449 F1 instrument coupled to a QMS 403 Aeolos Quadro mass spectrometer. The sample was loaded into a lidded alumina crucible, which was then evacuated and purged with nitrogen gas three successive times. The sample was then heated from 40 to 600 °C at a rate of 5 °C min^–1^ under a nitrogen gas flow (50 mL min^–1^). Mass and heat fluctuations due to the lidded alumina crucible were corrected. The evolved gas from the outlet was analyzed using mass spectrometry (MS), monitoring a mass-to-charge (*m*/*z*) ratio of 18, confirming the mass loss was attributable to water.

### 2.3 Magic-Angle Spinning Nuclear Magnetic Resonance (MAS NMR) Spectroscopy

Solid-state ^29^Si NMR spectra were acquired by using a Bruker Avance III spectrometer equipped with a wide-bore 9.4 T magnet, operating at a Larmor frequency of 79.44 MHz for ^29^Si. Samples were packed into a 4 mm (outside diameter) rotor and studied using a conventional Bruker HX magic-angle spinning (MAS) NMR probe with a spinning rate of approximately 14 kHz. Direct excitation spectra were obtained following a 90° pulse with a 120 s recycle interval. Chemical shifts are given relative to TMS, measured using a secondary standard of Q8M8 (octakis(trimethylsiloxy)silsesquioxane) at 11.3 ppm.

### 2.4 Powder X-ray Diffraction (PXRD)

Powder X-ray diffraction (PXRD) patterns were recorded at the Diamond Light Source synchrotron on beamline I11, the high-resolution PXRD beamline,^15^ operating at 15 keV with a precise wavelength of λ = 0.824407 Å, calculated from a Pawley fit on a silicon standard.

The zeolite sample was contained within a polyimide capillary (OD = 0.794 mm; ID = 0.694 mm), obtained from Cole Parmer, and supported at each end by a plug of glass wool. The capillary was inserted in a bespoke flow cell, which permits data to be recorded on solid samples experiencing dynamic liquid flow. In the flow cell setup, a syringe pump was used to flow a 20 mM CsNO_3_ solution through Teflon tubing to a reducing union, which joins the sample-containing capillary via a porous stainless-steel frit (10 μm pore diameter), as shown in a schematic in the Supporting Information (Figure S3). Throughout the experiment, the dynamic flow of fresh solution through the sample was maintained at a 0.05 mL min^–1^ rate. The solution was made by dissolving the appropriate amount of cesium nitrate (99.8%), obtained from Alfa Aesar, in ultrapure water (18.2 MΩ).

During the experiment, a PXRD pattern was recorded every 20 s on a Mythen position sensitive detector with a 10 s scan time followed by a 10 s delay to permit writing of the data. Each pattern was collected across the 2θ range 2.084–92.132° with a 0.004° step size. The sample was rocked through 4° in space about the capillary to ensure powder averaging during data collection. Each completed rocking motion, moving 4° anticlockwise and then 4° clockwise to return to the original position, lasted approximately 5 s. Accordingly, ca. two complete rocking motions took place during each recorded PXRD pattern. Data was collected over a period of 4.2 h while exchange took place. Under the experimental conditions, the solution concentration must be sufficiently high that appreciable exchange takes place over the entire probed period but not so high that significant exchange occurs between each 20 s interval in which each PXRD pattern is recorded. It was found that 20 mM CsNO_3_ satisfied this condition, but higher concentrations gave rise to significant exchange during the recording of the first few patterns.

### 2.5 Rietveld Refinement

Principal component analysis (PCA) in DAWN software^16^ was employed to monitor changes in the diffraction data to indicate when exchange commenced and to highlight areas of interest in the data set from which individual scans were then selected for Rietveld analysis. The former was necessary as there is a delay between when the syringe pump begins to inject the solution into the Teflon tubing in the experimental setup and when the solution reaches the sample-loaded capillary, PCA therefore enables the precise time at which the ion-exchange reaction begins to be identified by monitoring changes in the recorded data. A plot of the principal component as a function of scan number may be found in the Supporting Information (Figure S4).

Rietveld refinements were performed on selected PXRD patterns, across the 2θ range 4–70°, using GSAS-II software with a shifted-Chebyschev background and a pseudo-Voigt profile function with a Finger-Cox-Jephcoat asymmetry correction.^17^ The zero point was refined for the first pattern and then fixed in all subsequent scans. The lattice parameters, sample displacement perpendicular to the beam, the Gaussian Caglioti terms (U, V, and W), the Lorentzian crystallite size broadening term (X), Stephen’s microstrain broadening terms (Sxxx), and the axial divergence term (SH/L) were refined in all scans. Good fits were observed in all instances with weighted profile factors (*R*_wp_) spanning the range *R*_wp_ = 4.11–6.39%. A plot of *R*_wp_ as a function of log_10_(*t*), where *t* is the time in seconds that the PXRD pattern was recorded after the exchange commenced at *t* = 0, is contained in the Supporting Information (Figure S5).

The starting model for the crystal structure and the initial positions of the cation sites for both potassium and cesium were based on the structures reported by Kong et al.^8^ The atomic positions and isotropic thermal displacement parameters were refined for all atoms in the crystal structure. Moreover, the fractional occupancies for the extraframework cations, K^+^ and Cs^+^, and the water molecules in the structure have also been refined. In all refinements, these parameters were refined until convergence was achieved. As Si^4+^ and Al^3+^ are isoelectronic and occupy the same site in the structure, a single tetrahedral site (denoted T) encompassing both the Si^4+^ and Al^3+^ ions has been used, as it is conventional in the Rietveld analysis of zeolites. The T-O bond length was restrained at 1.65 ± 0.06 Å. For the water molecules, only the oxygen atom was included in the refined structure, as hydrogen atoms may not be identified from X-ray diffraction data. In the two refinements where the W5 site was identified, a restraint was applied to the W5–O1 and W5–O3 distances at 2.80 Å to prevent the W5 site from refining unreasonably close to these framework oxygen atoms. An equivalence was employed for all isotropic thermal displacement parameters on atoms of a given type. In the scan recorded at *t* = 15099 s, the thermal displacement parameter for the T atom was fixed at *U*_iso_ = 0.001 Å^2^, as refining led to a negative value with low magnitude.

A constraint was applied on the total sum of extraframework cations to match that which would be expected from the Si/Al ratio of the material (Si/Al = 2.28) obtained from quantitative ^29^Si MAS NMR spectroscopy. A further constraint was applied on refinements of the three scans recorded at 2470, 7057, and 11070 s to constrain the fractional occupancies for K3 (F_occ_(K3)) and Cs2 (F_occ_(Cs2)) such that 2(F_occ_(K3)) + (F_occ_(Cs2)) = 1.00, as in the absence of this constraint the total sum tended to refine to values between 1.06 and 1.09, whereas the maximum possible value is 1.00.

The results of the refinements, including the atomic positions, isotropic thermal displacement parameters, fractional occupancies, lattice parameters, and weighted and unweighted profile factors, may be found in the associated CIF.

## 3 Results

**Figure 2:**
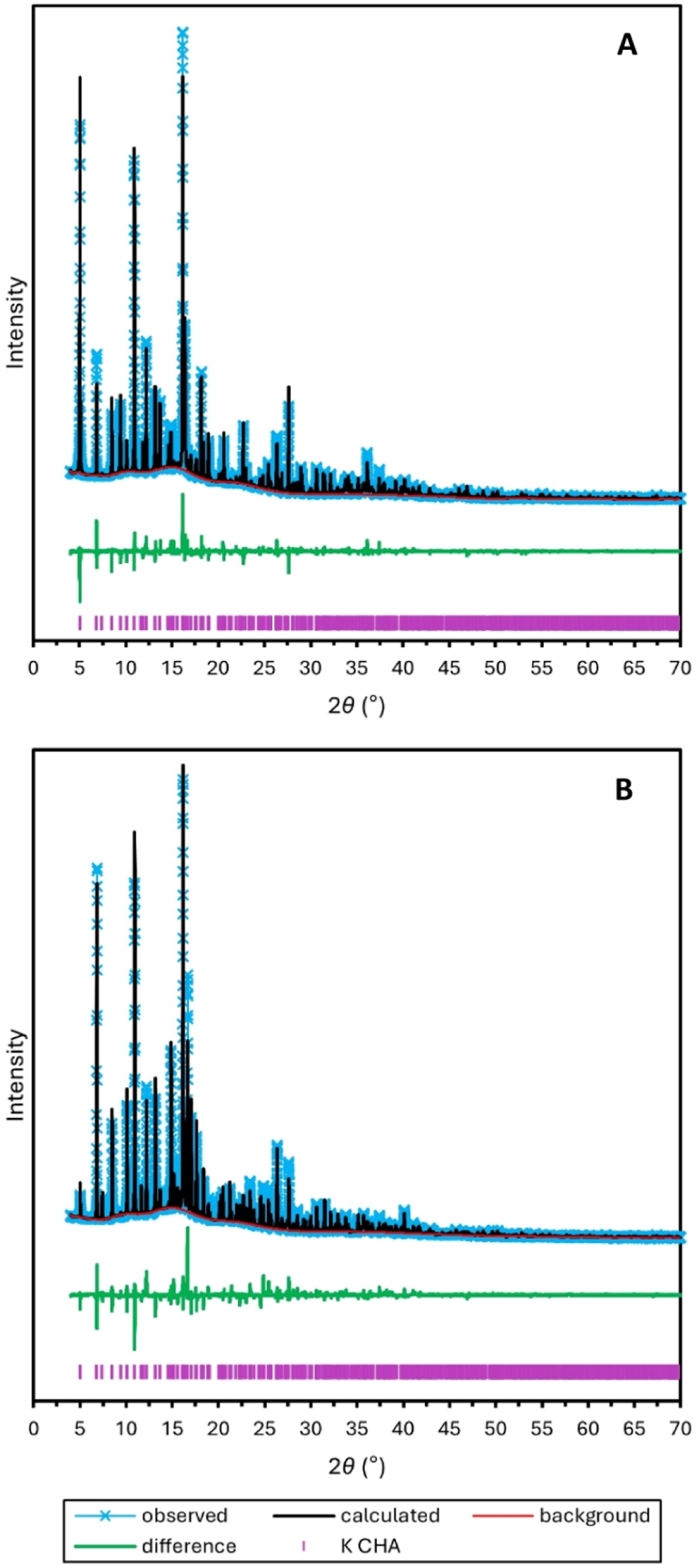
Rietveld refinements of K-CHA PXRD patterns recorded at *t* = 0 s (A) and 15099 s (B).

**Figure 3:**
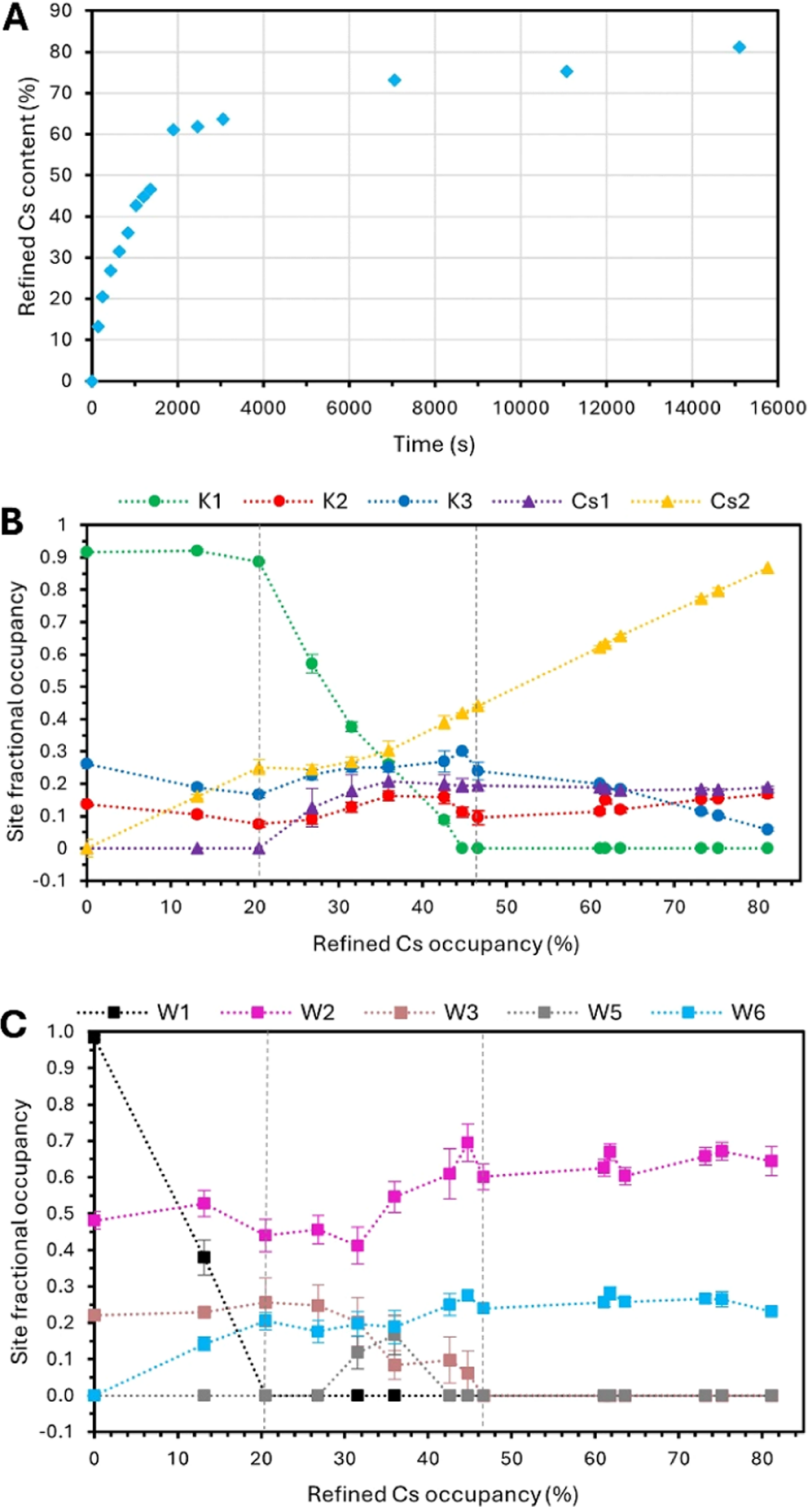
(A) Plot of the proportion of occupied extraframework cation sites containing Cs^+^ (%) as a function of time (s). (B) Plot of occupancies for the extraframework cation sites as a function of the refined proportion of the total extraframework sites occupied by Cs (%). Each different site is differentiated by color as indicated in the figure. (C). An equivalent plot to (B) for the fractional occupancies on the crystallographic water sites. In both (B) and (C), error bars are present where the magnitude exceeds the size of the marker and represent 3 times the standard deviation in the value calculated by GSAS-II. The dashed gray vertical lines demarcate the stages mentioned in the discussion.

*In situ* PXRD patterns have been recorded every 20 s as exchange proceeds between potassium chabazite (K-CHA) and aqueous Cs^+^ ions over the course of 4.2 h. Fifteen selected patterns from across this range, including the termini, have been analyzed by Rietveld refinement to reveal detailed mechanistic information about the spatial distribution of extraframework cations and water molecules within the crystal structure as the ion-exchange reaction proceeds. The 15 selected PXRD patterns are presented in Figure S7. Rietveld refinements of the terminal scans, recorded at *t* = 0 and 15,099 s, are presented in Figure 2. Based on the structural changes observed in the refined results, the exchange reaction may be divided into three distinct stages, which are each described in successive sections. Stage 1 covers the first 4 min of the reaction, *t* = 0–241 s, and the proportion of extraframework cations sites occupied by Cs^+^ rising from 0 to 21%. Stage 2 covers *t* = 241–1366 s and the Cs^+^ content rising from 21 to 46%, while Stage 3 covers *t* = 1366–15099 s and the Cs^+^ content increasing from 46 to 81%. A plot of the refined Cs^+^ content as a function of time is presented in Figure 3A. In the subsequent mechanistic discussion, the referenced fractional occupancies for the extraframework cation and water sites are presented in Figure 3B,C, respectively, as a function of the refined Cs^+^ content.

In addition to characterization by Rietveld refinement, the K-CHA employed in this study has also been characterized by thermogravimetric analysis (Figure S6), which indicates that there are 8.47 water molecules per formula unit. Moreover, a Si/Al ratio of 2.28 was calculated from the deconvoluted signals in the ^29^Si MAS NMR spectrum (Figures S1 and S2). Accordingly, the K-CHA employed in this study has the chemical formula: K_3.67_Si_8.33_Al_3.67_O_24_.8.47H_2_O. The chemical formulas at each probed stage of the *in situ* exchange, as determined by Rietveld refinement, are presented in Table S1.

### 3.1 Stage 1

Stage 1 spans the first 4 min of the ion-exchange reaction, at the end of which, 21% of the extraframework cations are Cs^+^ residing solely on the Cs2 site (Figure 3B). The outgoing K^+^ ions have mostly been lost from the K2 and K3 sites at the center of the cage, which are the origins of 17 and 75%, respectively, of the K^+^ exchanged out of the structure at this point. The final 8% has been exchanged with the K1 site.

In the refined structure of the starting K-CHA, three water sites are identified and correspond to the W1, W2, and W3 sites observed previously.^7,8^ The freely refined fractional occupancies at these three sites total 8.48 water molecules per unit cell, which is in excellent agreement with the value obtained from thermogravimetric analysis (Figure S6): 8.47 molecules per unit cell. The W4 site observed previously^7,8^ at (0.3068, −0.4189, 0.5020) was not found to have any occupancy in the starting material or in the patterns recorded during the exchange. In a previous report, the chabazite studied possessed a higher degree of hydration (10.7 water molecules per unit cell)^7^ than the chabazite employed in this study, which may rationalize the absence of the W4 site. A site close to W1 (0.500, 0.500, 0) but with reduced symmetry, denoted W1*, was reported by Kong et al.^8^ occurring at (0.498, 0.498, 0.101); however, no occupancy was observed at this site in the starting material in this study, but a higher occupancy was instead observed on the W1 site.

A significant rearrangement of the water molecules within the structure is observed by the end of Stage 1, as shown in Figure 3C. The fractional occupancy at the W1 site decreases from almost full, 0.984, to empty over this period. The W1 site is located at the center of the S8R aperture, which permits diffusion between adjoining cages in the material, as is necessary for ion exchange to occur. Occupancy at the W1 site is therefore incompatible with high levels of ion exchange occurring in the material and rationalizes the rapid loss of water from this site once exchange begins. Most of the water molecules displaced from the W1 site (83%) migrate to the W6 site, which is not occupied before ion exchange commences, but it is one of the two water environments expected in Cs-CHA along with the W2 site. A further 15% of the water molecules displaced from W1 migrate to the W3 site. There is also a decrease in the absolute values for the W2 fractional occupancy over this period; however, this variation is within the bounds of associated error. There is a reasonably high degree of associated error in some of the fractional occupancies on the water sites during the first two stages of exchange, which may be rationalized by inherent disorder on these sites while the exchange is taking place, as the *in situ* PXRD patterns have been recorded on a dynamic system.

### 3.2 Stage 2

Stage 2 spans the next 18.75 min of the reaction, from *t* = 241 to 1366 s, in which the proportion of extraframework cation sites occupied by Cs^+^ rises from 21 to 46%. The K1 site empties over this period, and 50% of these K^+^ cations lost from the site are exchanged out of the zeolite, with 22% being replaced by Cs1 and 28% replaced by Cs2. The remaining 50% of the K^+^ cations lost from the K1 site are not exchanged from the zeolite but are instead involved in a rearrangement, migrating to the K2 and K3 sites in the center of the cage, with 45% of the original K1 ions migrating to K3 and a further 5% migrating to K2.

During this period, the W3 site also empties, with 20% of the water molecules migrating to the W6 site and 30% migrating to the W2 site by the end of the period. The remaining 50% of the molecules from the site appear to be lost from the structure, and it may be possible that these lost water molecules coordinate with the departing potassium ions from the K1 site as they migrate to the zeolite surface. Alternatively, it is also possible that the water remains within the structure but is completely disordered and, therefore, does not contribute to the PXRD pattern. A new water position (W5) was identified in the refined structures of patterns recorded at *t* = 642 and 843 s and appears to be an intermediate site that is filled from water molecules migrating from W3. The presence of the W5 site in these structures was found to improve the agreement between calculated and observed intensity on several lower-intensity reflections, most notably (11̅ 1̅) and (111).

### 3.3 Stage 3

Stage 3 covers the final 3.82 h of the reaction, from *t* = 1366 to 15099 s, where the proportion of extraframework cation sites occupied by Cs^+^ increases from 46 to 81%. All incoming Cs^+^ resides on the Cs2 site, corresponding to 87% of K^+^ lost from the K3 site during this period. The remaining 13% of K^+^ ions lost from the K3 site migrate to the K2 site. The Cs1 site remains constant, within error, over the entire period. Exchange in this period is markedly slower than the previous stages and may be correlated with high occupancy on the Cs2 site, which resides in the center of the S8R apertures that permit diffusion through the structure. As occupancy at Cs2 increases, the ability of K^+^ ions to diffuse between adjacent cages and eventually out to the surface becomes increasingly impeded. The rate of the exchange reaction decreases significantly from *t* = 1906 s, at which point 62% of the Cs2 sites are occupied, demonstrating the rate-limiting impact of reduced diffusion in the material (Figure 3A). While 61% of the extraframework K^+^ ions are replaced by Cs^+^ in the first 31.8 min of the exchange reaction, it takes a further 219.9 min for the next 20% of the extraframework K^+^ ions to be replaced.

In this final stage, there is comparatively little variation in the water positions in the structure. The only water sites that are found to be occupied in this range are W2 and W6, which is in agreement with previous structural studies on cesium-containing chabazite samples.^8,10^ While some small variations are observed in the occupancies over the range, these are largely within the bounds of the associated error. The degree of error in the fractional occupancies is significantly lower when compared with those of previous stages, reflecting the slower exchange kinetics and resulting reduced disorder during this final stage of the reaction.

### 3.4 Kinetics

**Figure 4:**
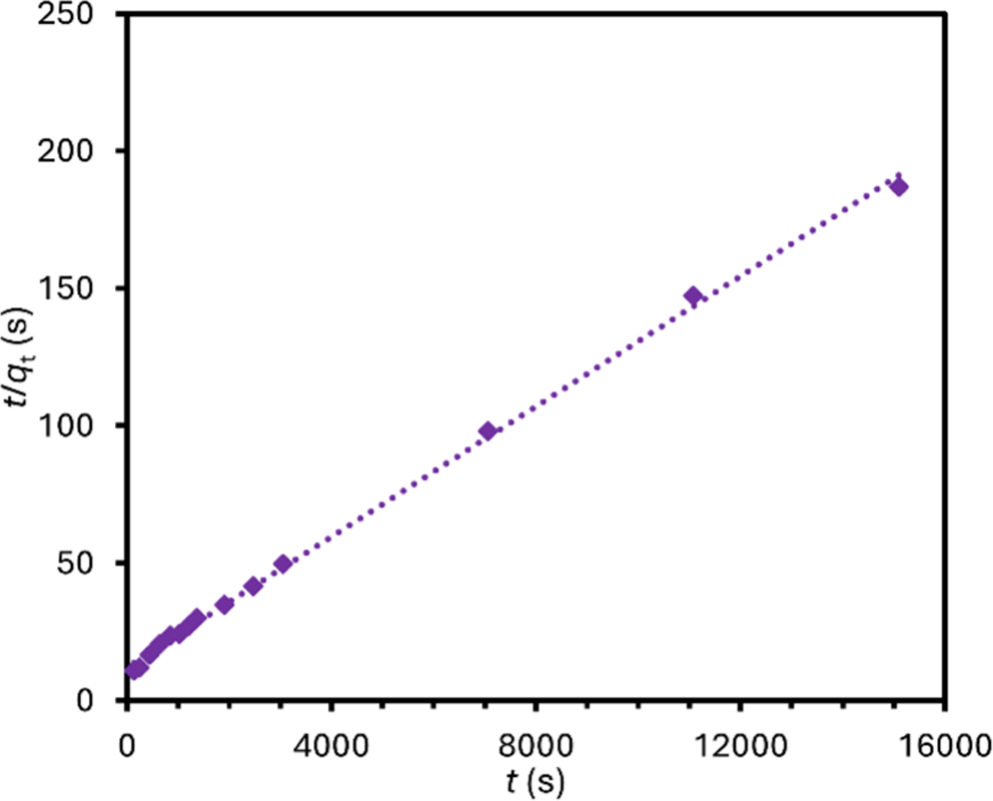
Plot of the linear pseudo-second-order kinetic model (*t*/*q_t_* vs *t*), where *q_t_* is the refined Cs content obtained from Rietveld refinements on PXRD data recorded at time (*t*) during the exchange reaction.

Several kinetic models have been employed to relate changes in the refined Cs^+^ content in the zeolite with time; the equations for these models as collated in a review by Gupta and Bhattacharya^18^ are presented in the Supporting Information (Table S2) along with the *R*^2^ value for the fits. While a pseudo-second-order kinetic model is found to best fit the data (*R*^2^ = 0.998) as plotted in Figure 4, the limitations of this model have been expounded by Simonin.^19^ The pseudo-second-order model does not account for intraparticle diffusion, which is a non-negligible consideration in ion-exchange reactions in nanoporous solids. Accordingly, good agreement between the data and the model is likely to be arbitrary in this case. Moreover, a pseudo-second-order model provides no additional mechanistic information beyond the information gleaned from the refined crystal structures.

### 3.5 Thermal Displacement Parameters

The thermal displacement parameters for the W sites, which have been grouped with an equivalence, increase from *U*_iso_ = 0.023(2) Å^2^ in K-CHA to 0.131(6) Å^2^ at the end of Stage 1, reflecting increased disorder on these sites once exchange commences. Elevated thermal displacement parameters are sustained throughout Stage 2, ranging from 0.110(6) to 0.179(9) Å^2^ but cover a reduced range in Stage 3: 0.062(7)–0.126(6) Å^2^. The reduction in values in Stage 3 intimates less disorder on these sites, as the rate of reaction also decreases in this period. However, the values remain significantly higher than in the original material before the exchange commenced, and this may be rationalized by continued disorder on these sites as the exchange reaction proceeds toward equilibrium. As the sample is immersed in an aqueous solution, these high thermal displacement parameters may also be associated with disorder, which is consequent upon dynamic exchange between water molecules within the zeolite and those in the solution.

Trends and variations indicative of the dynamic nature of the reaction are also observed in the thermal displacement parameters for the extraframework cations. There is little variation outside the bounds of error for the K^+^ thermal displacement parameter from the starting zeolite, *U*_iso_ = 0.0467(9) Å^2^, to the end of Stage 1, *U*_iso_ = 0.039(1) Å^2^, but a significant increase in this value is observed across Stages 2 and 3, spanning *U*_iso_ = 0.074(5)–0.18(6) Å^2^, indicating significant disorder on these sites as the K^+^ content continues to deplete. In contrast, the thermal displacement parameter for Cs^+^ is initially high with *U*_iso_ = 0.127(3) Å^2^ at the end of Stage 1. As the reaction proceeds and the Cs content continues to increase, the thermal displacement parameter for Cs^+^ decreases with values ranging from 0.0359(7) to 0.075(2) Å^2^ across Stages 2 and 3, indicating reduced disorder on these sites as the exchange proceeds.

While the dynamic nature of the ion-exchange reaction leads to inflated thermal displacement parameters for the extraframework species, the thermal displacement parameters for the framework sites possess conventional values and are unaffected by the disorder within the pores. Values for the T and framework O sites were, respectively, 0.0025(2) and 0.0262(6) Å^2^ in the original unexchanged zeolite, while the values over the course of the reaction span 0.0018(4)–0.0088(4) Å^2^ for the T site and 0.019(1)–0.043(1) Å^2^ for the framework O site.

## 4 Discussion

While *in situ* PXRD studies have been previously reported on inorganic ion exchangers, such as crystalline silicotitanate (CST)^11,12^ and zirconosilicate umbite,^13^ this present study is the first to probe ion exchange in a zeolite. A 7-fold enhancement in temporal resolution has been achieved in the present study, with 1 scan recorded every 20 s, compared with previous studies, which achieved 1 scan every 140 or 150 s.^11−13^ In studies on CST, only the extraframework cation positions and occupancies were refined, with water sites inferred from computational modeling and thermal displacement parameters and framework atomic positions fixed at predetermined values.^11,12^ In studies on the zirconosilicate, thermal displacement parameters were again fixed, but the framework and water positions were refined; however, the constraints employed likely underestimated the overall water concentration in the material. By contrast, in the present study, atomic positions and isotropic displacement parameters have been refined for all nonhydrogen atoms, as well as relevant fractional occupancies, revealing previously inaccessible structural details. The positions and fractional occupancies of crystallographic water sites have also been freely refined. The refinement of thermal displacement parameters provides a measure of disorder on the extraframework sites as exchange occurs and demonstrates the need to refine these parameters in *in situ* data sets collected during the exchange.

New mechanistic insights attained from analyzing the *in situ* data include the nonlinear changes in extraframework cation site occupancy as exchange progresses, the redistribution of K^+^ cations among established crystallographic sites during exchange, and how the rearrangement of intrapore water molecules from the sites observed in the K-form of the zeolite to the sites observed in the Cs-form takes place. Such intermediate phenomena have not previously been observed, as such information is naturally inaccessible by *ex situ* methods applied to the equilibrium crystal structures of exchanged zeolites.

Ultimately, the present *in situ* study improves the understanding of ion-exchange processes in zeolites, revealing the nonequilibrium intermediates by which ion exchange proceeds and demonstrating the complexity of these dynamic processes. This may have a significant influence on both the modeling of ion-exchange phenomena and the further study of ion exchange in zeolites, and other inorganic ion exchangers, by *in situ* diffraction methods.

## 5 Conclusions

By applying Rietveld refinement to *in situ* synchrotron PXRD data collected on potassium chabazite as it undergoes Cs-ion exchange, a time-resolved structural mechanism for this exchange reaction has been obtained. The relative temporal changes in fractional occupancies for extraframework cations reveal which sites are filled and replaced as exchange progresses, while the refined fractional occupancies for water molecules show the rearrangement that takes place within the pores as exchange proceeds. Based on the structural changes, the exchange reaction may be divided into three distinct stages.

A pseudo-second-order kinetic model gives good agreement in relating changes in the refined Cs^+^ content with time (*R*^2^ = 0.998). While the exchange reaction initially proceeds rapidly, the reaction rate decreases markedly once 61% Cs^+^ content is reached, correlating with high occupancy on the Cs2 site, centered in the S8R apertures that permit diffusion throughout the structure. It appears that high occupancy at the Cs2 site inhibits diffusion, exerting a rate-limiting impact on the reaction.

Ultimately, in this study, nonequilibrium crystal structures have been obtained from *in situ* PXRD data recorded on a dynamic system, giving novel mechanistic insights into an ion-exchange reaction occurring in a zeolite.

## Data Availability

All raw crystallographic data are embedded within the associated CIF file. The NMR research data underpinning this publication can be accessed at 10.17630/b1e57582-c20c-4e7a-bfbb-e241428ebac3.^20^ Any other data that support the findings of this study are available from the corresponding authors upon request.

## Supporting Information Available

^29^Si MAS NMR spectrum and deconvolution, flow cell schematic, principal component analysis plot, plot of *R*_wp_ as a function of log_10_*t*, TGA-MS plot, time-resolved PXRD patterns, tabulated refined chemical formulas, tabulated kinetic models, and quality of fits (PDF)
Crystal structures and raw crystallographic data (CIF)

## Supplementary Material

jp4c02145_si_001.pdf

jp4c02145_si_002.cif
