# Supplementary material for: Mechanistic Insights into Cs-Ion Exchange in the Zeolite Chabazite from In Situ Powder X-Ray Diffraction
Source: J Phys Chem C Nanomater Interfaces. 2024 May 29;128(23):9735–41. doi: 10.1021/acs.jpcc.4c02145 (PMC11182343; doi:10.1021/acs.jpcc.4c02145)
Supplement: Supplementary file 1 — jp4c02145_si_001.pdf [file jp4c02145_si_001.pdf]

Supporting Information

Mechanistic Insights into Cs-Ion Exchange in the Zeolite Chabazite from *In-Situ* Powder X-Ray Diffraction

Daniel S. Parsons[a*], Antony Nearchou[b], Ben L. Griffiths[c], Sharon E. Ashbrook[c], Joseph A. Hriljac[a,b**]

[a] Diamond Light Source Ltd, Harwell Science and Innovation Campus, Didcot, Oxfordshire, OX11 0DE, United Kingdom

[b] School of Chemistry, University of Birmingham, Edgbaston, Birmingham, West Midlands, B15 2TT, United Kingdom

[c] School of Chemistry, EaStCHEM and Centre of Magnetic Resonance, North Haugh, University of St Andrews, St Andrews, Fife, KY16 9ST, United Kingdom

*drdanparsons@gmail.com

**joseph.hriljac@diamond.ac.uk

Item	Page
Figure S1. ^{29}Si MAS NMR spectrum of K-CHA	S2
Figure S2. Deconvolution of the ^{29}Si MAS NMR spectrum of K-CHA	S2
Figure S3. Schematic of the flow cell arrangement and components.	S3
Figure S4. Plot of principal component analysis	S3
Figure S5. Plot of R_{wp} as a function of $\log_{10}t$	S4
Figure S6. TGA-MS plot.	S4
Figure S7. Time resolved PXRD patterns.	S5
Table S1. Refined chemical formulae.	S6
Table S2. Kinetic models and quality of fits against collected data.	S6

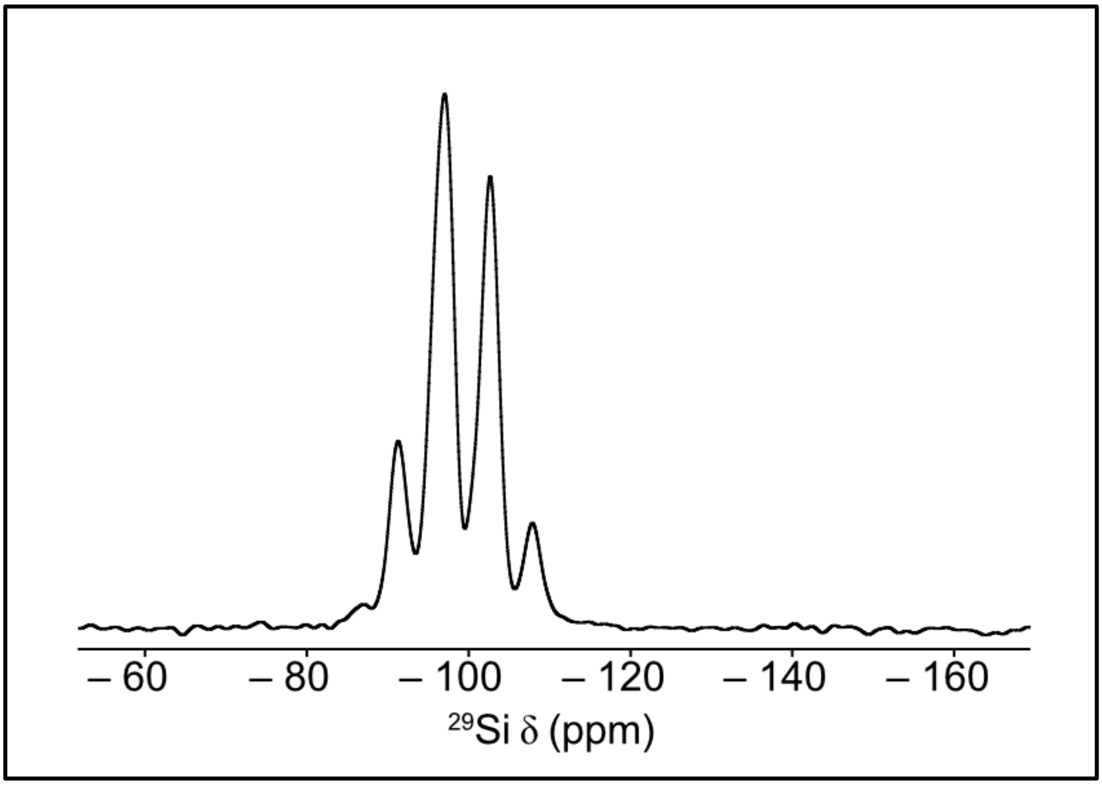

Figure S1. ^{29}Si MAS NMR spectrum of K-CHA.

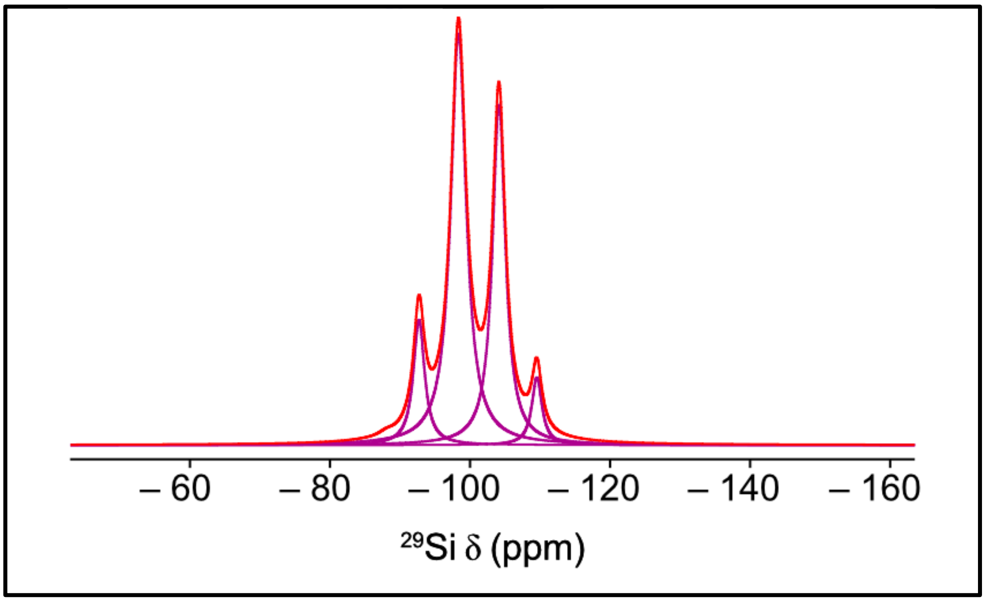

Figure S2. Deconvolution of the ^{29}Si MAS NMR spectrum of K-CHA.

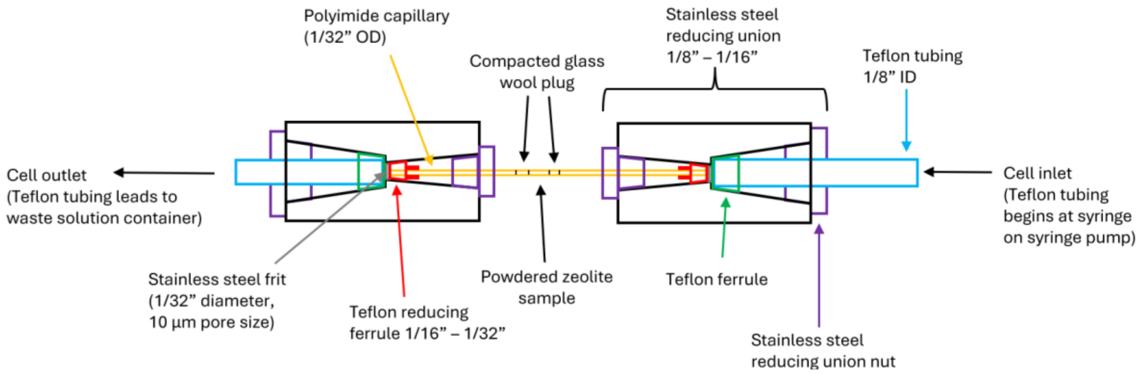

Figure S3. Schematic of the flow cell arrangement and components.

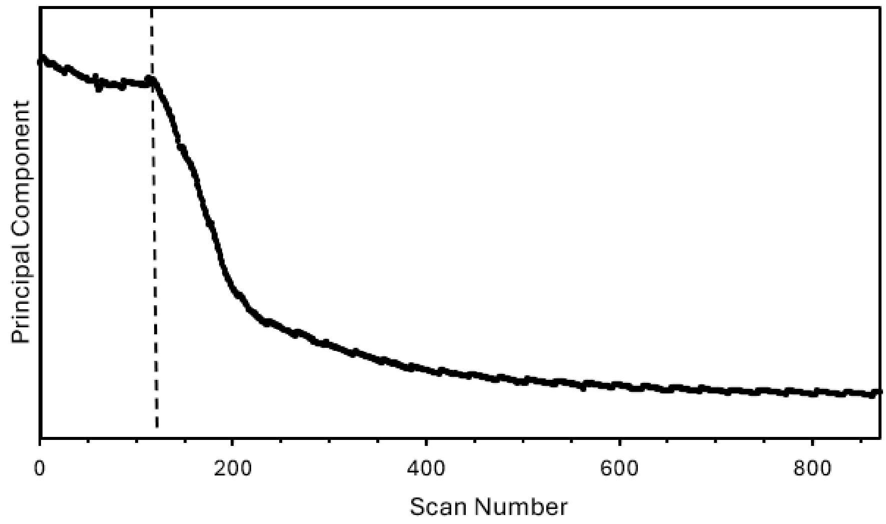

Figure S4. Plot of the principal component as a function of scan number, calculated in DAWN software for the 870 *in situ* PXRD patterns. The vertical dashed line shows when exchange commences.

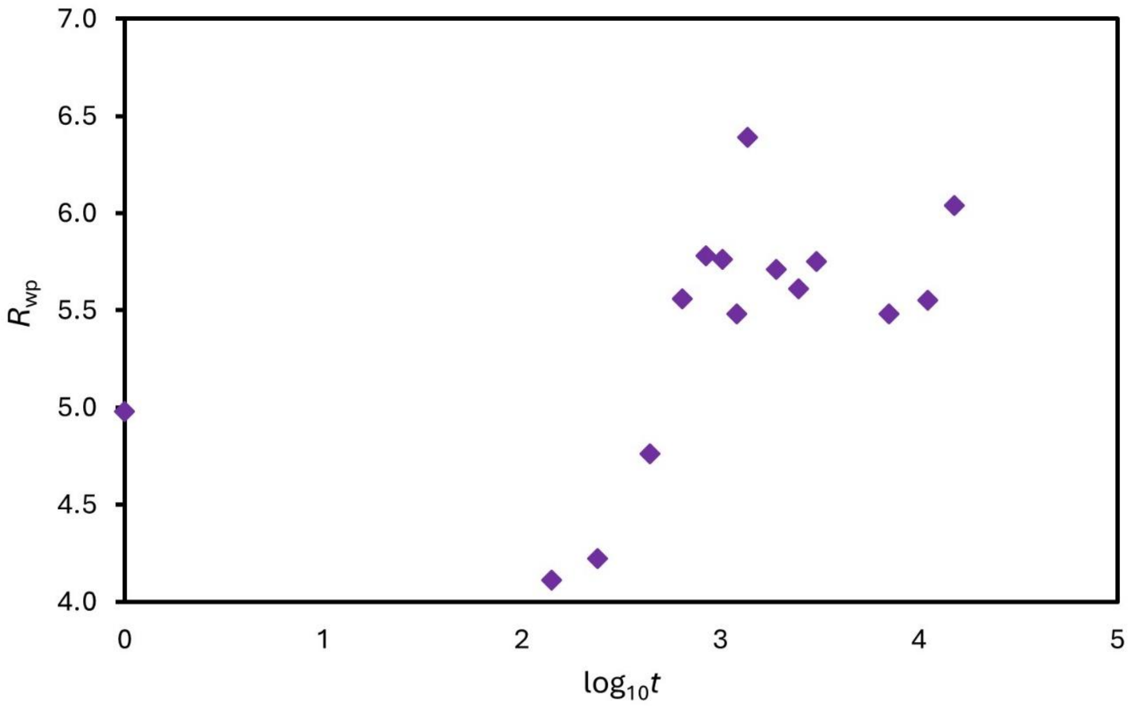

Figure S5. Plot of the weighted profile factor (R_{wp}) for the Rietveld refinements as a function of $\log_{10}t$, where t is the time that the PXRD pattern was recorded after exchange commenced at t = 0.

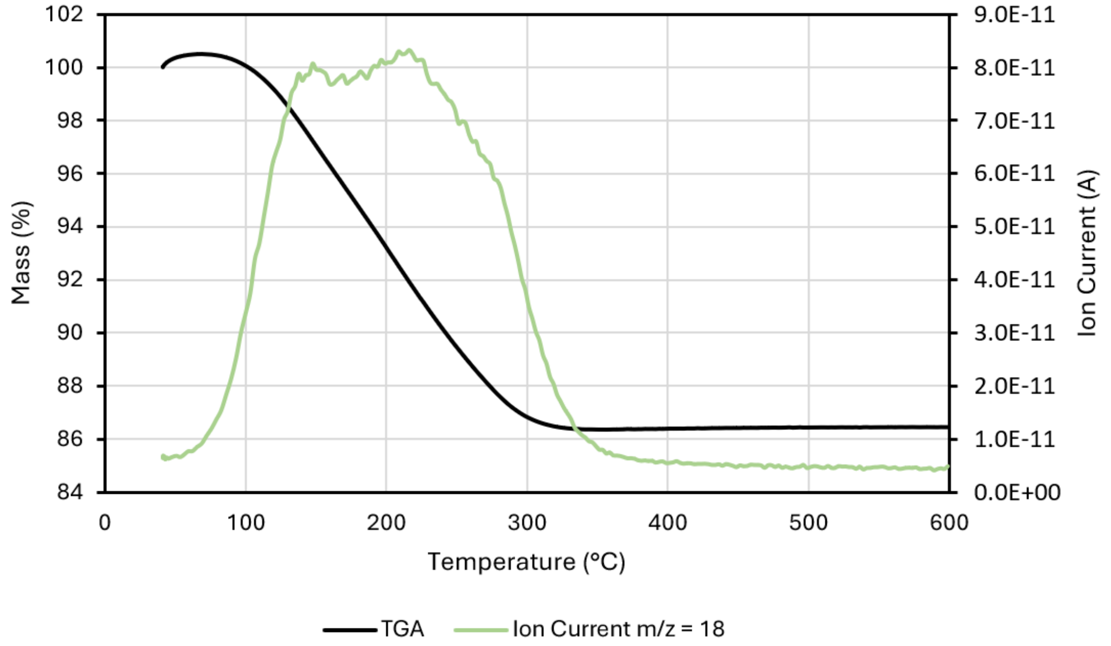

Figure S6. Thermogravimetric analysis on the potassium chabazite where the black line is a plot of the sample mass (%) as a function of temperature (°C), and the green line is a plot of ion current (A) registered by the mass spectrometer for m/z = 18 (i.e. water ions) as a function of temperature (°C).

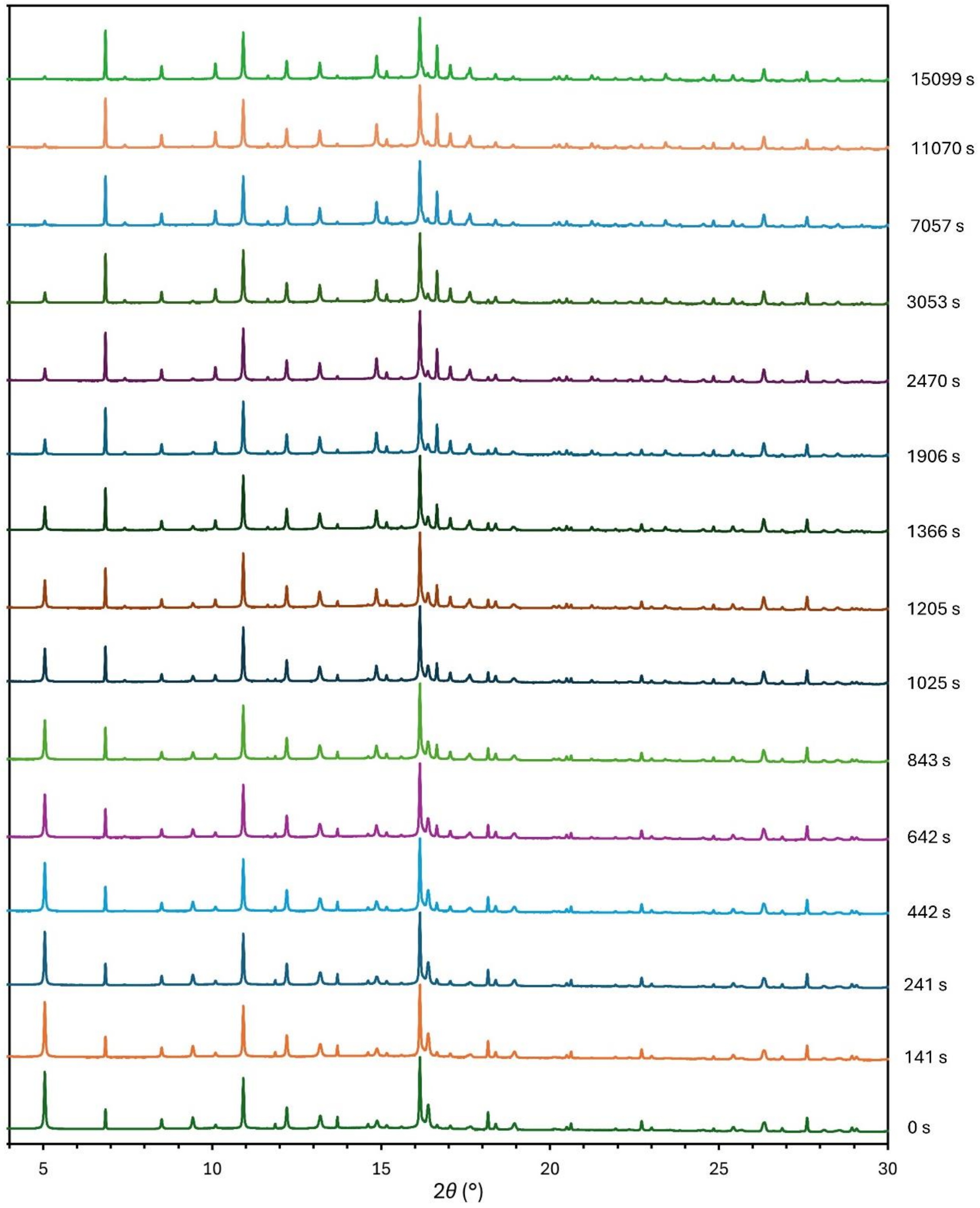

Figure S7. Time-resolved PXRD patterns that were refined in this study. The label denotes the time interval in seconds at which the pattern was recorded from when exchange commenced at t = 0 s.

Table S1. Refined chemical formulae for the PXRD patterns recorded at the given time interval in seconds from when exchange commenced at t = 0 s.

Time (s)	Refined chemical formula
0	$K_{3.67}Si_{8.33}Al_{3.67}O_{24}.8.48H_2O$
141	$K_{3.19}Cs_{0.48}Si_{8.33}Al_{3.67}O_{24}.8.76H_2O$
241	$K_{2.92}Cs_{0.75}Si_{8.33}Al_{3.67}O_{24}.8.17H_2O$
442	$K_{2.69}Cs_{0.98}Si_{8.33}Al_{3.67}O_{24}.7.81H_2O$
642	$K_{2.51}Cs_{1.16}Si_{8.33}Al_{3.67}O_{24}.7.97H_2O$
843	$K_{2.35}Cs_{1.32}Si_{8.33}Al_{3.67}O_{24}.7.54H_2O$
1025	$K_{2.11}Cs_{1.56}Si_{8.33}Al_{3.67}O_{24}.7.83H_2O$
1205	$K_{2.03}Cs_{1.64}Si_{8.33}Al_{3.67}O_{24}.8.21H_2O$
1366	$K_{1.98}Cs_{1.69}Si_{8.33}Al_{3.67}O_{24}.6.67H_2O$
1906	$K_{1.44}Cs_{2.23}Si_{8.33}Al_{3.67}O_{24}.6.83H_2O$
2470	$K_{1.40}Cs_{2.27}Si_{8.33}Al_{3.67}O_{24}.7.39H_2O$
3053	$K_{1.33}Cs_{2.34}Si_{8.33}Al_{3.67}O_{24}.6.70H_2O$
7057	$K_{0.99}Cs_{2.68}Si_{8.33}Al_{3.67}O_{24}.7.14H_2O$
11070	$K_{0.92}Cs_{2.75}Si_{8.33}Al_{3.67}O_{24}.7.19H_2O$
15099	$K_{0.69}Cs_{2.98}Si_{8.33}Al_{3.67}O_{24}.6.62H_2O$

Table S2. Kinetic models applied to adsorption at the solid-liquid interface as reported by Gupta and Bhattacharyya[19]. The terms in the equations are defined as follows: t, time (s); q_e, final Cs content; q_t, Cs content at given time t; k_1, first order adsorption rate coefficient; k_2, second order adsorption rate coefficient; α, initial adsorption rate (s^{-2}); β, desorption co-efficient (s^{-1}); D_c, intracrystalline diffusivity. The intraparticle diffusion model does not include the final data point ($q_t = q_e$) as this gives a non-numerical y value.

Model	R^2	Linear equation
Lagergren first order	0.875	$\ln(q_e - q_t) = \ln q_e - k_1 \cdot t$
Pseudo second order	0.998	$t/q_t = 1/(k_2 q_e{}^2) + (1/q_e) \cdot t$
Elovich	0.969	$q_t = \beta \ln(\alpha\beta) + \beta \ln t$
Intraparticle diffusion[a]	0.887	$\ln(1 - q_t/q_e) = \left(-\pi^2 D_c/r^2\right) \cdot t + \ln\left(6/\pi^2\right)$
